# Patient characteristics and healthcare utilisation among Danish patients with chronic conditions: a nationwide cohort study in general practice and hospitals

**DOI:** 10.1186/s12913-020-05820-3

**Published:** 2020-10-26

**Authors:** Anders Damgaard Møller Schlünsen, David Høyrup Christiansen, Ulrich Fredberg, Peter Vedsted

**Affiliations:** 1 Diagnostic Centre, University Research Clinic for Innovative Patient Pathways, Department of Clinical Medicine, Silkeborg Regional Hospital, Aarhus University, Silkeborg, Denmark; 2 Department of Occupational Medicine, Regional Hospital West Jutland, University Research Clinic, Herning, Denmark; 3 Department of Clinical Medicine, HEALTH, Aarhus University, Aarhus, Denmark; 4 Department of Public Health, Research Unit for General Practice, Aarhus University, Aarhus, Denmark

**Keywords:** Delivery of health care, Chronic disease, Healthcare utilisation, Chronic obstructive pulmonary disease, Inflammatory bowel disease, Chronic liver disease, Atrial fibrillation, Congestive heart failure, Denmark

## Abstract

**Background:**

The complexity of caring for patients with chronic conditions necessitates new models of integrated care to accommodate an increasing demand. To inform the development of integrated care models, it is essential to map patients’ use of healthcare resources. In this nationwide registry-based cohort study, we describe and compare patient characteristics and healthcare utilisation between Danish patients with chronic conditions in general practice follow-up and in hospital outpatient follow-up.

**Methods:**

On 1 January 2016, we identified 250,402 patients registered in 2006–2015 with a hospital diagnosis of atrial fibrillation/flutter, congestive heart failure, chronic liver disease, inflammatory bowel disease or chronic obstructive pulmonary disease. By linkage to national social and health registries, patient characteristics and 12-month healthcare utilisation were extracted. Incidence rates of health care utilisation were compared between patients with chronic conditions in general practice follow-up and patients in hospital outpatient follow-up using negative binomial regression.

**Results:**

Across all five conditions, the largest proportions of patients were in general practice follow-up (range = 59–87%). Patients in hospital outpatient follow-up had higher rates of exacerbation-related admissions (adjusted incidence rate ratio (IRR) range = 1.3 to 2.8) and total length of stay (IRR range = 1.2 to 2.2). For these five conditions, all-cause admissions and lengths of stay, general practice daytime and out-of-hours contacts, and municipal home nursing contacts were similar between follow-up groups or higher among patients in general practice follow-up. The exception was patients with chronic obstructive pulmonary disease, where patients in hospital outpatient follow-up had higher utilisation of healthcare resources.

**Conclusions:**

Patients in general practice follow-up accounted for the largest proportion of total healthcare utilisation, but patients in hospital outpatient follow-up were characterised by high exacerbation rates. Enhanced integration of chronic care may be of most benefit if patients in general practice follow-up are targeted, but it is also likely to have an impact on exacerbation rates among patients in hospital outpatient follow-up.

## Background

Chronic conditions are increasingly a global cause of morbidity and mortality [1]. The increasing prevalence [2] is primarily driven by ageing populations [3] and is leading to an increased demand for healthcare services [4]. Complex conditions and multimorbidity entail patient pathways involving multiple specialties across sectors [5] and challenge healthcare systems in terms of meeting the demand while ensuring continuity of care and high patient satisfaction.

The provision of healthcare in Denmark is divided mainly between general practitioners (GPs), public hospitals and municipalities. Overall quality of the Danish system is considered satisfactory [6], but fragmentation is a threat to chronic care due to a lack of continuity between sectors [7]. This is further complicated by a high degree of specialisation in hospitals and a shortage of GPs [8]. Danish chronic care is intended to be guided by the principles of the Chronic Care Model (CCM) and risk stratification [6]. This means that the majority of patients with chronic conditions have regular follow-up appointments in general practice, while a small proportion characterised by a moderate to high degree of complications have additional follow-up appointments in the specialised hospital outpatient setting. The place of follow-up is intended to be guided by a risk stratification based on clinical parameters pertaining to the specific condition. The place of follow-up may change correspondingly with changes in these parameters. In Denmark, risk stratification is well implemented for chronic obstructive pulmonary disease (COPD) where the place of follow-up is guided by the Global Initiative for Chronic Obstructive Lung Disease (GOLD) criteria. Formal criteria are lacking for other chronic conditions and the division of care has yet to be empirically investigated as a first step for improving chronic care delivery between general practice and hospitals.

Integrated care refers to the ability of healthcare systems to deliver coordinated and cohesive care within and between providers [9]. Integrated care has been suggested to reduce healthcare costs [10] and improve the quality of care [11]. Results have shown favourable outcomes in patient satisfaction, perceived quality of care and access to services [12], while results on healthcare utilisation and costs are mixed [9, 13, 14]. However, integrated care interventions are complex [15], and neither interventions nor outcomes may necessarily be reproduced in contexts other than the original [12]. Thus, to inform the development and adoption of integrated care interventions, a detailed mapping of healthcare delivery is necessary [16].

The aim of this study was to describe and compare patient characteristics and healthcare utilisation in Danish patients with chronic conditions in general practice follow-up and hospital outpatient follow-up.

## Methods

### Design and setting

The study was a nationwide registry-based cohort study in Denmark, which has 5.8 m citizens and is characterised as a social welfare state [7]. The Danish healthcare sector is tax financed and provides free coverage for all Danish citizens. Some services are based on co-payment (e.g. dental care, physiotherapy, psychologists and prescriptive medication). Administration of the Danish healthcare system is undertaken by the state, five geographical regions and 98 municipalities. Each region is responsible for running and coordinating public hospitals and primary healthcare services. Municipalities are responsible for eldercare, social psychiatry and health promotion [8]. The primary entry point to the Danish healthcare system is the GPs, who act as gatekeepers to specialised services and coordinate different health services to ensure progress and coherence in the patient pathways [8].

In 2005, the Danish National Board of Health published recommendations based on the CCM to improve integrated care in Denmark. This led to several initiatives to promote patient self-management, decision support, health information technologies and delivery system design [6, 7]. Despite implementation of these initiatives, poor coherence in patient pathways crossing sectors is still considered a weakness of the Danish healthcare system. Danish regions and municipalities have a key role in ensuring integration of care. Every fourth year, each region and the corresponding municipalities negotiate the content of the collaboration on six mandatory areas (hospitalisation and discharge, rehabilitation, devices and aids, prevention and health promotion, mental health and follow-up on adverse events). However, the GPs are not formally a part of the planning of these agreements.

### Registries

All data for this study were retrieved from Danish national health and social registries. Danish citizens have a unique civil registration number which makes it possible to link information between registries [17]. The following registries were utilised:

#### Health registries

1) The Danish National Patient Register (DNPR): Since 1977 the DNPR has collected information about all in- and outpatient contacts in all Danish public hospitals [18, 19]. The registry is characterised as highly complete.

2) The Danish National Health Service Register (DNHSR): This registry contains information about all general practice daytime and out-of-hours services contacts (i.e. face-to-face contacts, home visits, telephone contacts and e-mail contacts) since 1990 [20]. The DNHSR is based on invoices used for reimbursing primary care providers. Clinical information (e.g. reason for contact and diagnosis) is not available in the registry.

3) Home nurse registry: The home nurse registry contains information about municipal home nurse contacts [21, 22]. A contact constitutes a visit in the patient’s own home performed by a municipal nurse. The registry contains information about the date of the contact, the recipient and if the contact was acute or scheduled. The registry is yet to be validated. Outcomes based on these data should be interpreted with caution.

#### Social registries

1) The Danish civil registration system (CRS): The CRS contains information about age, gender, vital status, immigration, emigration and residence [17].

2) Family type: This registry contains information about the household status, based on information about persons living on a particular address and registered interrelationships [23].

3) Citizens living in nursing homes: This registry contains information about individuals moving to and from nursing homes [22, 24].

4) Personal income: the registry has information about the personal income in a calendar year [25]. The income is calculated as the sum of all incomes (except for any rental value of own accommodation) before deducting labour-market contributions and pension contributions.

5) Socioeconomic classification (version 13): Based on information about source of income, this registry contains information about the occupational status [26].

6) Highest obtained educational level: The registry contains information about the highest obtained educational level based on the International Standard Classification of Education 1997 (ISCED97) [27].

### Study population

The index date was 1 January 2016. The cohort consisted of all Danish citizens alive on this date who were registered in the DNPR between 2006 and 2015 with a primary or secondary inpatient or outpatient hospital International Classification of Disease, 10th revision (ICD-10) diagnosis of inflammatory bowel disease (IBD), chronic liver disease (CLD), COPD, congestive heart failure (CHF) or atrial fibrillation (AF) (See Appendix 1.a for specific ICD-10 diagnoses). The inclusion of these particular diagnoses was guided by a new Danish model of integrated care aiming to reduce acute healthcare utilisation in patients with these conditions who have regular follow-up appointments in a hospital outpatient clinic [28].

In the present study, we distinguished between patients with regular follow-up appointments only in general practice and patients with follow-up appointments in both general practice and a hospital outpatient clinic. For the sake of brevity, we shall refer to patients in general practice follow-up and patients in hospital outpatient follow-up. Patients in the latter group were identified by the presence of an ongoing hospital outpatient follow-up pathway in the DNPR on the index date which had lasted for at least 6 months. Patients with no ongoing hospital outpatient follow-up pathway on the index date were considered to be in general practice follow-up while a hospital outpatient follow-up pathway that had lasted less than 6 months on the index date was assumed to be a temporary assignment to GP care.

### Follow-up

Follow-up time in this study was 12 months. All information during this period was retrieved from the described registries.

### Variables

#### Patient characteristics

For this study to have an explorative approach, we included multiple variables to describe personal, socio-economic and disease characteristics. Specifically, patient characteristics included gender, age, nursing home residence status, household status (Appendix 1.b), gross income, occupational status (Appendix 1.c), highest level of education (Appendix 1.d), Charlson co-morbidity index (CCI) [29] including all hospital diagnoses registered from 2006 to 2015. The ICD-10 diagnosis codes used for identifying the qualifying condition were omitted in the CCI calculation.

#### Hospital utilisation outcomes

Data on hospital utilisation from the DNPR included information about acute hospital admissions and total acute length of stay (LOS) as well as scheduled outpatient visits. Based on the assigned ICD-10 diagnosis, we distinguished admissions and LOS by exacerbation admissions, other admissions and all-cause admission (Appendix 1.e). Likewise, we distinguished outpatient visits by condition-related visits, other visits and all-cause visits based on the primary ICD-10 diagnosis code (Appendix 1.a).

#### General practice utilisation

Data on general practice utilisation included daytime contacts (Appendix 1.f) and out-of-hours contacts (Appendix 1.g). Out-of-hours contacts were not reported for patients living in the Capital Region of Denmark. This region had established a non-GP based out-of-hours service from which data are not collected in the DNHSR.

#### Home nurse visits

We included scheduled and acute municipal nurse visits delivered in the patient’s own home (Appendix 1.h). This outcome was not reported for patients living in nursing homes.

### Statistical analysis

Descriptive data on categorical and dichotomous outcomes are reported by percentages. For continuous and count data, we report mean and standard deviation (SD) or median and interquartile interval (IQI), depending upon data distributions.

Healthcare utilisation data are reported as incidence rates per person per year. For calculating rates of outcomes that could not occur when a patients was hospitalised (e.g. a new admission or a GP contact), we subtracted any days spent in hospital in the follow-up period from the total time at risk [30]. To assess differences in outcomes between patients in general practice and hospital follow-up, we used a negative binomial regression model. We report adjusted incidence rate ratios (IRR) with corresponding 95% confidence intervals (CI) based on robust standard errors to accommodate multiple observations per patient. Adjusted models included group status, gender, age, nursing home residence status, educational level and CCI.

A patient in hospital follow-up was censored if this ended during this study. In contrast, a patient in general practice follow-up was censored if the patient switched to hospital outpatient follow-up due to the qualifying chronic condition during the study period. Data were analysed using STATA 15 (StataCorp LP, College Station, TX, USA).

### Ethics

According to Danish law, studies that are based on registry data alone are not required to obtain permission from the regional ethics committees, which was confirmed by The Central Denmark Region Committees on Health Research Ethics (REF: 1–10–72-148-19). The Danish data protection agency approved the study (REF: 2009-41-3471).

## Results

A total of 250,402 patients were included: 83% had one, 15% had two and 2% had three or more of the included five chronic conditions. Patients with AF comprised the largest group (*n* = 114,795) and CLD the smallest (*n* = 12,398) (Table 1). The majority of patients with these five conditions were in general practice follow-up (range = 59–87%). Primary school as the highest obtained educational level was more often observed among patients in general practice follow-up. For patients with COPD, AF and CLD, levels of CCI were comparable between patients in general practice and hospital outpatient follow-up. Among patients with IBD and CHF, patients in general practice follow-up had slightly higher levels of CCI.

**Table 1:** Characteristics of 250,402 patients with chronic conditions in general practice or hospital follow-up

	AF (***n*** = 114,795)		CHF(***n*** = 56,858)		CLD(***n*** = 12,398)		COPD(***n*** = 69,247)		IBD(***n*** = 45,160)	
	Hospital follow-up (***n*** = 15,595, 14%)	GP follow-up (***n*** = 99,200,86%)	Hospital follow-up (***n*** = 8426,15%)	GPfollow-up (***n*** = 48,432,85%)	Hospital follow-up (***n*** = 3308,27%)	GP follow-up (***n*** = 9090,73%)	Hospital follow-up (***n*** = 9245,13%)	GPfollow-up (***n*** = 60,002,87%)	Hospital follow-up (***n*** = 18,610,41%)	GP follow-up (***n*** = 26,550,59%)
**Female**	37%	42%	30%	39%	55%	46%	55%	53%	54%	55%
**Age**, mean (SD)	71.1 (11.0)	72.9 (12.6)	67.1 (13.6)	73.1 (12.7)	60.8 (13.1)	60.6 (13.6)	70.8 (10.2)	70.6 (11.9)	48.9 (16.6)	52.0 (17.8)
**Household status**										
Married or cohabiting	64%	57%	60%	51%	53%	46%	48%	47%	68%	65%
Single	36%	43%	40%	49%	47%	54%	51%	52%	32%	35%
**Nursing home residents**	2%	5%	2%	6%	2%	4%	4%	5%	< 1%	1%
**Gross income in 2015 (DKK),** mean (SD)	315,925 (562,682)	286,118 (520,366)	282,663 (455,070)	258,366 (463,668)	259,943 (243,346)	248,741 (198,318)	237,236 (618,625)	234,166 (229,007)	347,175 (364,015)	318,224 (921,789)
**Occupational status**										
Working	22%	18%	22%	14%	23%	19%	9%	13%	59%	51%
Unemployed	2%	2%	6%	3%	11%	12%	4%	5%	7%	8%
Early retiree	4%	4%	11%	8%	20%	24%	14%	12%	6%	8%
Retired	71%	75%	60%	75%	42%	40%	72%	69%	19%	26%
Others	1%	1%	2%	1%	4%	4%	1%	1%	9%	7%
**Highest obtained educational level**										
Primary education	32%	39%	37%	44%	35%	39%	49%	50%	22%	28%
Secondary education or vocational training	40%	39%	43%	39%	44%	43%	38%	37%	44%	44%
Higher education	27%	22%	20%	17%	21%	19%	12%	13%	34%	28%
**Months since first diagnosis,** mean (SD)	61.9 (42.5)	57.1 (39.1)	54.2 (42.2)	55.6 (39.4)	64.4 (51.6)	58.7 (45.6)	56.0 (39.5)	46.3 (40.8)	99.7 (62.7)	92.5 (61.6)
**CCI distribution**										
None	38%	37%	32%	25%	51%	45%	47%	48%	78%	71%
Low	42%	41%	43%	47%	36%	37%	37%	36%	18%	22%
Moderate	14%	15%	17%	19%	10%	12%	12%	11%	3%	5%
High	6%	7%	7%	9%	4%	6%	4%	5%	1%	2%
**CCI,** median (IQI)	1 (0–2)	1 (0–2)	1 (0–2)	1 (0–3)	0 (0–2)	1 (0–2)	1 (0–2)	1 (0–2)	0 (0–0)	0 (0–1)

Numbers may not add up to 100% due to rounding. Abbreviations: *AF* atrial fibrillation, *CHF* congestive heart failure, *CLD* chronic liver disease, *COPD* chronic obstructive pulmonary disease, *IBD* inflammatory bowel disease, Hospital; patients in long-term hospital outpatient follow-up, *GP* patients in general practice follow-up, *SD* standard deviation, *DKK* Danish kroner, *CCI* Charlson co-morbidity index, *IQI* interquartile interval

**Fig. 1:**
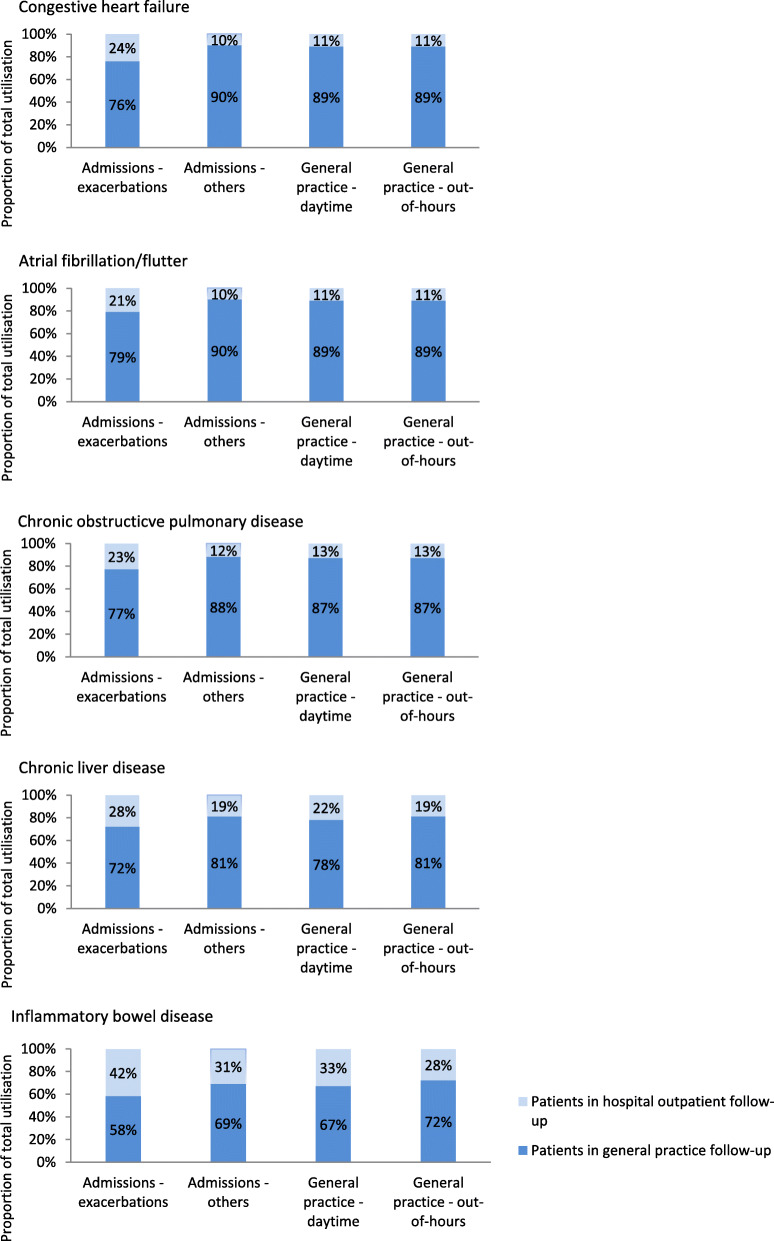
Proportions of total utilisation by patients with chronic conditions in general practice or hospital follow-up

Results for hospital utilisation are shown in Table 2. Rates of all-cause acute admissions were comparable between patients in general practice and hospital outpatient follow-up. The exception was COPD patients, where patients in hospital outpatient follow-up had higher rates of all-cause admissions (adjusted IRR = 1.42, CI = 1.36–1.48). Across all five conditions, patients in hospital outpatient follow-up had higher rates of exacerbation admissions. However, admissions and outpatient visits were more often caused by conditions other than the index condition. Patients in general practice follow-up accounted for the largest proportion of all exacerbation admissions (Fig. 1).

**Table 2:** Hospital utilisation among patients with chronic conditions in general practice or hospital follow-up

		AF			CHF			CLD			COPD			IBD		
Outcome	Follow-up group	Crude rates^**a**^	IRR	Adj IRR (95% CI)^**b**^	Crude rates^**a**^	IRR	Adj IRR (95% CI) ^**b**^	Crude rates^**a**^	IRR	Adj IRR (95% CI) ^**b**^	Crude rates^**a**^	IRR	Adj IRR (95% CI) ^**b**^	Crude rates^**a**^	IRR	Adj IRR (95% CI) ^**b**^
**Acute admissions**																
Exacerbation admissions	Hospital	.15	2.11	1.95 (1.80–2.11)	.11	2.40	2.61 (2.26–3.01)	.20	1.46	1.44 (1.16–1.78)	.66	2.32	2.41 (2.26–2.56)	.08	1.31	1.29 (1.14–1.47)
	GP	.07			.05			.16			.29			.06		
Other admissions	Hospital	.67	.83	.89 (.86–.93)	.82	.76	.89 (.85–.94)	.83	.76	.81 (.74–.88)	.91	1.05	1.08 (1.03–1.13)	.35	.78	.93 (.88–.97)
	GP	.78			1.05			1.10			.88			.44		
All-cause admissions	Hospital	.81	.94	1.01 (.97–1.05)	.93	.83	.97 (.92–1.03)	1.04	.84	.88 (.81–.97)	1.58	1.33	1.42 (1.36–1.48)	.42	.84	.99 (.94–1.05)
	GP	.86			1.10			1.26			1.17			.51		
**Total LOS**																
Exacerbation admissions	Hospital	.18	1.22	1.22 (1.04–1.44)	.49	1.67	2.13 (1.66–2.74)	.97	1.47	1.49 (1.07–2.08)	2.96	2.06	2.27 (2.07–2.49)	.22	1.20	1.27 (1.04–1.55)
	GP	.15			.26			.75			1.28			.18		
Other admissions	Hospital	2.12	.70	.78 (.71–.85)	3.08	.70	.91 (.83–1.00)	2.95	.72	.80 (.68–.93)	3.29	1.06	1.14 (1.04–1.24)	.72	.58	.82 (.72–.93)
	GP	2.78			3.91			3.86			3.00			1.13		
All-cause admissions	Hospital	2.29	.73	.82 (.76–.89)	3.57	.76	1.01 (.92–1.11)	3.92	.84	.91 (.78–1.05)	6.25	1.37	1.49 (1.40–1.60)	.94	.66	.93 (.83–1.04)
	GP	2.94			4.18			4.61			4.27			1.32		
**Outpatient visits**																
Condition-related visits	Hospital	2.58	8.76	8.76 (8.32–9.21)	2.63	7.98	7.32 (6.88–7.78)	2.08	3.91	3.87 (3.47–4.33)	2.38	7.68	7.68 (7.27–8.11)	1.49	2.81	2.75 (2.60–2.91)
	GP	.36			.43			.61			.33			.64		
Other visits	Hospital	4.34	.94	.98 (.95–1.02)	5.37	.90	.88 (.84–.93)	4.27	.82	.86 (.79–.95)	4.79	.98	.96 (.92–1.01)	2.36	.77	.85 (.81–.89)
	GP	4.56			6.15			5.37			4.91			3.16		
All-cause visits	Hospital	6.92	1.47	1.59 (1.54–1.64)	8.00	1.33	1.35 (1.30–1.41)	6.36	1.14	1.22 (1.13–1.32)	7.17	1.42	1.46 (1.40–1.52)	3.85	1.11	1.23 (1.19–1.28)
	GP	4.92			6.58			5.97			5.24			3.80		

^a^Crude incidence rates are counts of the outcome per patient per year.

^b^ Adjusted by gender, age, nursing home residence status, Charlson co-morbidity and highest obtained educational level. The GP follow-up group is the reference group.

Abbreviations: *AF* atrial fibrillation, *CHF* congestive heart failure, *CLD* chronic liver disease, *COPD* chronic obstructive pulmonary disease, *IBD* inflammatory bowel disease, *IRR* incidence rate ratio, *Adj*. adjusted, *95% CI* 95% confidence interval, Hospital; patients in long-term hospital outpatient follow-up, *GP* patients in general practice follow-up. *LOS* length of stay

Results for general practice and municipal home nursing utilisation are shown in Table 3. General practice daytime utilisation was similar between patients in general practice and hospital outpatient follow-up. However, COPD patients in hospital outpatient follow-up had more general practice daytime contacts than patients in general practice follow-up (adjusted IRR = 1.07, CI = 1.05–1.09). Rates of general practice out-of-hours contacts tended to be lower among patients in hospital outpatient follow-up, except for COPD patients (adjusted IRR = 1.47, CI = 1.33–1.62).

**Table 3:** Primary care utilisation among patients with chronic conditions in general practice or hospital follow-up

		AF			CHF			CLD			COPD			IBD		
Outcome	Follow-up group	Crude rates^**a**^	IRR	Adj IRR (95% CI)^**b**^	Crude rates^**a**^	IRR	Adj IRR (95% CI)^**b**^	Crude rates^**a**^	IRR	Adj IRR (95% CI)^**b**^	Crude rates^**a**^	IRR	Adj IRR (95% CI)^**b**^	Crude rates^**a**^	IRR	Adj IRR (95% CI)^**b**^
**General practice**																
**Daytime**																
Face-to-face consultations	Hospital	9.84	1.01	1.03 (1.01–1.04)	8.71	1.00	1.04 (1.01–1.06)	6.33	1.00	.99 (.94–1.04)	7.73	.95	.94 (.92–.97)	4.64	.88	.95 (.93–.97)
	GP	9.91			8.78			6.36			8.10			5.29		
Home consultations	Hospital	.13	.50	.63 (.53–.74)	.12	.37	.66 (.55–.80)	.09	.56	.59 (.41–.84)	.30	1.28	1.51 (1.32–1.73)	.02	.30	.75 (.51–1.11)
	GP	.25			.30			.16			.24			.06		
Telephone contacts	Hospital	5.00	.92	.98 (.96–1.00)	5.28	.82	.96 (.93–.99)	5.15	.87	.88 (.84–.93)	8.38	1.20	1.20 (1.17–1.23)	2.86	.80	.92 (.90–.95)
	GP	5.43			6.39			5.95			7.00			3.58		
Mail contacts	Hospital	2.93	.83	1.00 (.97–1.04)	2.98	.71	1.00 (.95–1.05)	2.55	.82	.89 (.81–.98)	3.56	1.09	1.10 (1.05–1.16)	1.69	.83	.94 (.90–.98)
	GP	3.43			3.99			3.14			3.27			2.01		
All	Hospital	17.91	.94	1.00 (.99–1.02)	17.10	.87	1.00 (.98–1.02)	14.12	.91	.92 (.89–.96)	20.00	1.07	1.07 (1.05–1.09)	9.21	.84	.94 (.92–.95)
	GP	19.02			19.47			15.60			18.61			10.94		
**Out-of-hours**^**c**^																
Telephone contacts	Hospital	.43	.79	.89 (.83–.95)	.47	.66	.90 (.81–1.00)	.58	.75	.84 (.69–1.01)	1.00	1.33	1.41 (1.24–1.61)	.33	.77	.87 (.81–.94)
	GP	.52			.66			.77			.77			.42		
Home consultations	Hospital	.19	.61	.76 (.69–.82)	.24	.51	.81 (.72–.91)	.27	.73	.75 (.61–.92)	.78	1.60	1.80 (1.60–2.03)	.07	.55	.82 (.70–.96)
	GP	.30			.41			.36			.50			.12		
Face-to-face consultations	Hospital	.18	1.15	1.13 (1.06–1.21)	.18	1.19	1.06 (.96–1.16)	.19	1.05	1.11 (.94–1.31)	.23	1.02	1.03 (.93–1.14)	.21	.91	.90 (.84–.98)
	GP	.16			.15			.18			.23			.23		
All	Hospital	.80	.77	.88 (.84–.93)	1.89	.66	.91 (.84–1.00)	1.04	.78	.86 (.73–1.00)	2.02	1.38	1.47 (1.33–1.62)	.61	.78	.88 (.82–.93)
	GP	.97			1.23			1.31			1.50			.77		
**Municipal home nurse**^**d**^																
Acute visits	Hospital	.55	.61	.72 (.59–.87)	.64	.47	.71 (.60–.83)	.61	.61	.67 (.47–.96)	1.23	1.19	1.39 (1.16–1.66)	.10	.28	.48 (.30–.77)
	GP	.87			1.26			.93			1.07			.31		
Scheduled visits	Hospital	28.31	.64	.68 (.59–.79)	27.80	.50	.79 (.66–.95)	30.19	.72	.79 (.63–1.01)	52.16	1.09	1.31 (1.16–1.47)	3.97	.33	.65 (.46–.92)
	GP	42.06			57.48			41.90			49.06			12.33		

^a^Counts of the outcome per patient per year.

^b^ Adjusted by gender, age, place of residence, Charlson co-morbidity and highest obtained educational level. The GP follow-up group is the reference group.

^c^ GP out-of-hours utilisation analyses do not include patients from the Capital Region of Denmark because this region has a non-GP based out-of-hours service

^d^Municipal home nurse utilisation analyses do not include patients who were nursing home residents.

Abbreviations: *AF* atrial fibrillation, *CHF* congestive heart failure, *CLD* chronic liver disease, *COPD* chronic obstructive pulmonary disease, *IBD* inflammatory bowel disease, *IRR* incidence rate ratio, *Adj*. adjusted, *95% CI* 95% confidence interval, Hospital; patients in hospital outpatient follow-up, *GP* patients in general practice follow-up

Rates of scheduled and acute municipal nurse contacts in the patient’s own home were higher for patients in general practice follow-up, except for COPD patients, where patients in hospital outpatient follow-up had more acute municipal nurse contacts (adjusted IRR = 1.39, CI = 1.16–1.66) and scheduled municipal nurse contacts (adjusted IRR = 1.31, CI = 1.16–1.47).

## Discussion

The majority of patients were in general practice follow-up only. Consequently, these patients contributed the most to exacerbation admissions and all-cause admissions, as well as general practice daytime and out-of-hours utilisation. Rates of admissions and LOS due to exacerbations were higher among patients in hospital outpatient follow-up than in patients in general practice follow-up. Except for COPD patients, adjusted IRRs for all-cause admissions were the same in patients in general practice and hospital follow-up. Patients followed only in general practice tended to have a lower educational level.

### Limitations and strengths

This study was based on a nationwide cohort of patients and is therefore less prone to selection bias due to social class or other confounding factors. Moreover, we have full follow-up, and the registries used in this study are considered highly complete. However, it is a weakness that we were unable to include out-of-hours contacts for patients in the Capital Region. Satisfactory positive predictive values have been obtained in earlier studies for the included chronic diagnoses, ranging from 65 to 100% [31–37]. However, our ability to identify condition-related hospital activity is diluted if diagnoses are unspecific or other than the relevant chronic diagnosis. A Danish study found that a third of all registered diagnoses in a joint ED were unspecific [38], and this may have caused us to underestimate exacerbations rates.

Our definition of patients in hospital follow-up was based on a minimum duration of 6 months to exclude patients in short-term diagnostic evaluations. Still, using an arbitrary minimum duration to determine the place of follow-up introduces a risk of selection bias. Some of the patients included as being primarily in general practice follow-up had been in contact with the hospital regarding their condition and reviewing electronic patient records would likely reveal that a proportion of these should belong to the hospital outpatient follow-up group. As a concept, the dichotomisation of patients as being in general practice or hospital outpatient follow-up is a simplification. Many patients will be in contact with various parts of the healthcare system during the course of their condition. This will tend to underestimate the actual differences between the two groups.

The analyses included adjustment for many known confounders that could have biased the healthcare utilisation outcomes. One important residual confounder we were not able to account for is the severity of the chronic condition. A higher proportion of the patients with the most severe conditions may be in hospital follow-up, which we also found indications of. Nevertheless, we can not rule out residual confounding caused by clinical differences that we were unable to adjust for.

### Interpretation of findings

In 2006, Vedsted and Olesen introduced the concept of chronic care risk stratification in Denmark [39]. They described that optimally, more than 90% of all patients should receive care in general practice. Our findings indicate that nearly this proportion of patients was in general practice follow-up; however, the number was lower for patients with IBD. This may partially be explained by the fact that entering our cohort required a hospital contact due to one or more of the relevant diagnoses. This omits the group of patients who did not receive hospital care because of their chronic condition.

This study shows that many patients with COPD are already followed up in general practice and that COPD patients in hospital follow-up may be those in highest need for hospital interventions. Thus, the Danish initiative to substitute hospital-based follow-up for COPD with general practice follow-up [40] may have unexpected consequences.

Our results showed that COPD patients in hospital outpatient follow-up seemed to be characterised by higher healthcare utilisation and poorer socio-economic outcomes compared to COPD patients in general practice follow-up. For all other included conditions, patients in hospital outpatient follow-up had, in contrast, less healthcare utilisation and better socio-economic indicators than patients in general practice follow-up. To the best of our knowledge, Danish risk stratification has been better and more widely implemented for COPD patients compared to the other patient groups included in this study. Our results may indicate that adopting functioning risk stratification in chronic care reduces social inequalities regarding who is granted access to specialised care when the place of follow-up is determined by objective criteria. When the boundaries of care division are unclear, this may permit socially advantaged patients to negotiate their way to specialised care, while the disadvantaged may not receive the care they should. Future healthcare planning may prioritise implementation of risk stratification as an element of integrated chronic disease management programmes to improve the delivery of integrated chronic care and to counteract social inequalities.

We found higher rates of exacerbation admissions among patients in hospital follow-up compared to patients in general practice follow-up. This is consistent with prior research [41] and the principle of risk stratification in Danish chronic care [6, 39]. This finding may support that integrated care interventions targeting exacerbations are implemented for patients in hospital outpatient follow-up. Qualitative studies have investigated what patients with chronic conditions find important in the delivery of integrated chronic care [42, 43]. Above all, patients prioritised a flexible and accessible system with continuity of care that was adapted to patient demands. A Danish study investigated healthcare professionals’ perspectives on barriers and enablers for integration of care across the primary and secondary care sector [44]. The study suggested that 24-h access to a helpline service for both patients and GPs could be an enabler for integrated care. We have earlier reported preliminary results for such clinic: a 24-h access outpatient clinic for patients with chronic conditions [28]. This clinic provided patients in hospital follow-up with round the clock telephone access in case of exacerbation. The results indicated reduced acute healthcare utilisation in both the primary and secondary sectors. The study was, however, conducted as a before and after study, and controlled studies are needed to determine whether the reductions can be attributed to the intervention [45].

Considering that patients in general practice follow-up accounted for the majority of all exacerbations and other outcomes of healthcare utilisation, future models of integrated care may rely on population-based approaches targeting the general practice population to benefit the majority of patients with chronic conditions and potentially lead to the greatest reductions in total healthcare utilisation. This echoes earlier studies suggesting that integrated chronic care should be population based [46]. The tendency to focus on preventable admissions among patients in hospital follow-up, i.e. high-risk patients, may therefore only lead to insignificant reductions in total acute healthcare utilisation and have a minimum of relevance.

A Danish study of barriers for integrated chronic care found that healthcare professionals considered poor accessibility and communication with hospital specialists a main obstacle [47]. Such a shortcoming is in conflict with basic recommendations for chronic care [39]. These include hospitals being available to GPs and delivering prompt specialist advice [48]. One-stop outpatient clinics may also serve as a valuable tool for clarification and early diagnosis [49, 50]. Integration of care may enable GPs to manage more care situations and minimise the need for hospital referrals. Bearing in mind a high rate of multimorbidity, health outcomes may be improved by maximising the proportion of care delivered by the GP, who has a generalist perspective [51].

The results of this study are most likely only transferable to countries with a healthcare sector organised in a fashion comparable with the Danish. This pertains particularly to the division of care responsibility between primary and secondary care, where the Danish healthcare system is conceptually based on a strong primary care sector to fulfil the concept of caring at the lowest efficient cost level. Generalisability of our results from 2016 to the present could be hampered by a significant change in the total healthcare utilisation since the study period. However, although we do not have data for the specific five chronic conditions included in this study, publicly available Danish registries of total national hospital utilisation suggested a slight reduction of admissions and bed days while the number of outpatient visits increased between 2016 and 2018 [52]. This indicates a movement towards substituting in-hospital activity with outpatient-based alternatives and this tendency would likely also be seen if our analyses were replicated with data from 2019 to 2020.

## Conclusion

A majority of patients with specific chronic diagnoses were in general practice follow-up only. These patients accounted for the greatest proportion of total healthcare utilisation. Relative rates of all-cause admission, all-cause LOS, general practice contacts and municipality contacts were comparable between follow-up groups or higher among patients in general practice follow-up, except for COPD patients, where the opposite was observed. In contrast, exacerbation admissions and LOS occurred at higher rates among patients in hospital outpatient follow-up. However, for these conditions, our results indicate that admissions were more often due to other causes than their primary chronic condition. Our findings support that integrating chronic care in patients in general practice follow-up may benefit the largest group of patients, who also account for the greatest proportion of total healthcare utilisation. For patients in hospital outpatient follow-up, integrated care may aim to reduce the high rates of exacerbation.

## Data Availability

The data that support the findings of this study are available from Statistics Denmark but restrictions apply to the availability of these data, which were used under license for the current study, and so are not publicly available. Data are however available from the authors upon reasonable request and with permission of Statistics Denmark.

## Appendix

**Table 4:** Identification of patients with specific chronic conditions and condition related outpatient visits

Registry (sector)	Diagnosis	Codes
DNPR (Hospital)		ICD-10
	IBD	K50*-K51*
	CLD	K658I, K702*, K703*, K704*, K711*, K717*, K72*, K73*, K74*, K754*, K761*, DK766*, K767, I85*
	COPD	J44, J440,J441 or J449
	CHF	I110, I130, I132, I420, I426, I427, I428*, I429, I500*, I501* or I509
	AF	I48*

**Table 5:** Identification of household status

Registry	Categories	Codes
Statistics Denmark		
	Single	5,9–10
	Married or cohabiting	1–4,7–8

**Table 6:** Identification of occupational status

Registry	Categories	Codes
Statistics Denmark		
	Working	110–114, 120, 131–135, 139
	Unemployed	210,220,230
	Early retiree	321
	Retired	322**–**323
	Others	410, 420, 310

**Table 7:** Identification of highest level of education

Registry	Categories	Codes
AFSP4E		
	Primary education	1*, 2*
	Secondary education or vocational training	3A*, 3C*, 4*
	Higher education	5*, 6*, 7*, 8*

**Table 8:** Identification of exacerbation related acute admissions

Registry (sector)	Diagnosis	ICD-10 codes
DNPR (Hospital)		
	IBD	K50*, K51*, R634, R10*, K30*, R64*, K20*, K21*, K221*, K25*, K26, K27, K28*, K29*, K315*, K566*, K625*, K630, K631*, K632*, K633, E43*, E44*, E46*, K908*, K909
	CLD	K65*, K702*, K703*, K704*, K711*, K717*, K72*, K73*, K74*, K754*, K761*, K766*-K767, I85*, I864*, I982, R17*, R18*, C22*
	COPD	J44*, J96*, J13*, J14*, J15*, J16*, J17*, J.18*
	CHF	I110, I130, I132, I420, I426, I427, I429, I50*
	AF	I48*

**Table 9:** Identification general practice daytime utilisation

Registry (sector)	Categories	Remuneration codes
DNHSR (GP)		
	Face-to-face consultation	0101
	Home consultation	0421, 0431, 0441, 0451, 0461, 0491
	E-mail contacts	0105
	Telephone contacts	0201

**Table 10:** Identification of general practice out-of-hours contacts

Registry (sector)	Categories	Remuneration codes
DNHSR (GP)		
	Telephone	0501
	Face-to-face consultation	0101
	Home consultation	0102, 0471

**Table 11:** Identification of municipal home nurse contacts

Registry (sector)	Categories	Codes
Statistics Denmark- Home nurse (Municipality)		
	Scheduled	1
	Acute	2

## Abbreviations

AF: Atrial fibrillation/flutter
CCI: Charlson comorbidity index
CCM: The Chronic care model
CHF: Congestive heart failure
CI: 95% confidence interval
CLD: Chronic liver disease
COPD: Chronic obstructive pulmonary disease
CRS: Danish Civil Registration System
DNHSR: Danish National Health Services Register
DNPR: Danish National Patient Register
ED: Emergency department
GOLD: Global Initiative for Chronic Obstructive Lung Disease
GP: General practitioner
IBD: Inflammatory bowel disease
ICD-10: International Classification of Disease, 10th revision
IQI: Interquartile interval
IRR: Incidence rate ratio
ISCED97: International Standard Classification of Education 1997
LOS: Length of stay
SD: Standard deviation
